# Supplementary material for: Ventromedial prefrontal cortex stimulation enhances memory and hippocampal neurogenesis in the middle-aged rats
Source: eLife. 2015 Mar 13;4:e04803. doi: 10.7554/eLife.04803 (PMC4381300; doi:10.7554/eLife.04803)
Supplement: Supplementary file 1. — The tables show the total exploratory duration during the acquisition phase, short- and long-term memory retention intervals in the novel-object recognition test for animal experiments of comparisons between the young and middle-aged rats (A), acute stimulation (B, C), and chronic stimulation (D, E) studies. Indication: *, significant difference from the sham rats. DOI: http://dx.doi.org/10.7554/eLife.04803.012 [file elife04803s001.doc]

**Supplementary File 1**

(A)

| **NOR Testing Phases** | **Age Group** | **Exploratory Duration**  **(Mean ± S.E.M.)** | **Effects** | |
| --- | --- | --- | --- | --- |
| **Acquisition Phase** | Young | 13.72 ± 1.904 | t_(28)_= −0.742, p= n.s. | Exploration:  F_(2,52)_=28.076, p<0.001.  Group:  F_(1,26)_= 0.097, p= n.s.  Group x Exploration:  F_(2,52)_= 2.492, p= n.s. |
|  | Middle-aged | 15.75 ± 1.728 |  |  |
| **Short-term memory** | Young | 23.32 ± 1.528 | t_(28)_= −0.725, p= n.s. |  |
|  | Middle-aged | 25.17 ± 2.121 |  |  |
| **Long-term memory** | Young | 23.41 ± 1.597 | t_(28)_= 1.526, p= n.s. |  |
|  | Middle-aged | 19.42 ± 2.144 |  |  |

(B)

| **NOR Testing Phases** | **High-Frequency**  **Stimulation Group** | **Exploratory Duration**  **(Mean ± S.E.M.)** | **Effects** | |
| --- | --- | --- | --- | --- |
| **Acquisition Phase** | 50 μA | 19.25 ± 3.98 | F_(4,40)_= 1.509, p= n.s. | Exploration:  F_(2,74)_=25.218, p<0.001.  Group:  F_(4,37)_=2.614, p= n.s.  Group x Exploration:  F_(8,74)_=2.164, p= 0.040. |
|  | 100 μA | 22.13 ± 3.25 |  |  |
|  | 200 μA | 14.70 ± 2.57 |  |  |
|  | 400 μA | 18.86 ± 1.08 |  |  |
|  | Sham | 14.42 ± 2.00 |  |  |
| **Short-term memory** | 50 μA | 30.00 ± 2.15 | F_(4,39)_= 3.656, p= 0.013 |  |
|  | 100 μA | 31.88 ± 2.65* |  |  |
|  | 200 μA | 26.50 ± 2.00 |  |  |
|  | 400 μA | 22.33 ± 3.56 |  |  |
|  | Sham | 21.50 ± 2.05 |  |  |
| **Long-term memory** | 50 μA | 19.50 ± 2.00 | F_(4,38)_= 2.914, p= 0.034 |  |
|  | 100 μA | 23.75 ± 2.11 |  |  |
|  | 200 μA | 23.00 ± 3.37 |  |  |
|  | 400 μA | 10.40 ± 2.73 |  |  |
|  | Sham | 15.92 ± 2.81 |  |  |

**(C)**

| **NOR Testing Phases** | **Low-Frequency**  **Stimulation Group** | **Exploratory Duration**  **(Mean ± S.E.M.)** | **Effects** | |
| --- | --- | --- | --- | --- |
| **Acquisition Phase** | 50 μA | 15.64 ± 2.03 | F_(4,50)_= 0.478, p= n.s. | Exploration:  F_(2,90)_=43.759, p<0.001.  Group:  F_(4,45)_=1.444, p= n.s.  Group x Exploration:  F_(8,90)_=1.956, p=n.s. |
|  | 100 μA | 14.00 ± 1.56 |  |  |
|  | 200 μA | 17.08 ± 1.96 |  |  |
|  | 400 μA | 17.25 ± 3.50 |  |  |
|  | Sham | 14.42 ± 2.00 |  |  |
| **Short-term memory** | 50 μA | 26.00±0.93 | F_(4,47)_= 1.564, p= n.s. |  |
|  | 100 μA | 22.36±3.54 |  |  |
|  | 200 μA | 29.83±1.93 |  |  |
|  | 400 μA | 27.29±3.38 |  |  |
|  | Sham | 21.50±3.49 |  |  |
| **Long-term memory** | 50 μA | 19.80 ± 2.69 | F_(4,48)_= 1.848, p= n.s. |  |
|  | 100 μA | 23.00 ± 3.46 |  |  |
|  | 200 μA | 24.25 ± 2.15 |  |  |
|  | 400 μA | 15.71 ± 2.93 |  |  |
|  | Sham | 15.92 ± 2.81 |  |  |

**(D)**

| **NOR Testing Phases**  **(no-HFS prior to testing)** | **Chronic**  **Stimulation Group** | **Exploratory Duration**  **(Mean ± S.E.M.)** | **Effects** | |
| --- | --- | --- | --- | --- |
| **Acquisition Phase** | vmPFC HFS | 22.40 ± 1.73 | t_(15)_= 1.531, p= n.s. | Exploration:  F_(2,30)_=9.061, p=0.001.  Group:  F_(1,15)_=7.861, p=0.013.  Group x Exploration:  F_(2,30)_=4.873, p=0.015. |
|  | Sham | 18.71 ± 1.46 |  |  |
| **Short-term memory** | vmPFC HFS | 29.25 ± 3.68* | t_(16)_= 2.430, p= 0.027 |  |
|  | Sham | 16.00 ± 1.97 |  |  |
| **Long-term memory** | vmPFC HFS | 18.27 ± 1.95 | t_(15)_= 1.533, p= n.s. |  |
|  | Sham | 14.00 ± 1.06 |  |  |

**(E)**

| **NOR Testing Phases**  **(HFS prior to testing)** | **Chronic**  **Stimulation Group** | **Exploratory Duration**  **(Mean ± S.E.M.)** | **Effects** | |
| --- | --- | --- | --- | --- |
| **Acquisition Phase** | vmPFC HFS | 14.89 ± 0.81 | t_(14)_= 1.272, p= n.s. | Exploration:  F_(2,30)_=8.505, p=0.001.  Group:  F_(1,15)_=16.721, p=0.001.  Group x Exploration:  F_(2,30)_=4.974, p=0.014. |
|  | Sham | 13.43 ± 0.78 |  |  |
| **Short-term memory** | vmPFC HFS | 25.60 ± 2.06* | t_(15)_= 2.684, p= 0.017 |  |
|  | Sham | 16.00 ± 3.12 |  |  |
| **Long-term memory** | vmPFC HFS | 21.50 ± 1.93* | t_(15)_= 4.221, p= 0.001 |  |
|  | Sham | 10.00 ± 1.70 |  |  |
