# Supplementary material for: Ventromedial prefrontal cortex stimulation enhances memory and hippocampal neurogenesis in the middle-aged rats
Source: eLife. 2015 Mar 13;4:e04803. doi: 10.7554/eLife.04803 (PMC4381300; doi:10.7554/eLife.04803)
Supplement: Supplementary file 2. — The tables show the total exploratory duration for both identical object 1 and 2 during the acquisition phase for animal experiments of comparisons between the young and middle-aged rats (A), acute stimulation (B), and chronic stimulation (C) studies. DOI: http://dx.doi.org/10.7554/eLife.04803.013 [file elife04803s002.doc]

**Supplementary File 2**

**(A)**

| **NOR Testing** | **Groups** | **Exploratory Duration**  **(Mean ± S.E.M.)** | | **Effects** |
| --- | --- | --- | --- | --- |
|  |  | **Object 1** | **Object 2** |  |
| **Acquisition Phase** | Young | 7.83 ± 1.28 | 5.89 ± 1.14 | t_(17)_= 1.294, p= n.s. |
|  | Middle-aged | 8.42 ± 1.63 | 7.33 ± 1.10 | t_(5)_= 0.498, p= n.s. |

**(B)**

| **NOR Testing**  **(Acquisition Phase)** | **Groups** | **Exploratory Duration**  **(Mean ± S.E.M.)** | | **Effects** |
| --- | --- | --- | --- | --- |
|  |  | **Object 1** | **Object 2** |  |
| **High-Frequency Stimulation** | 50 μA | 8.50 ± 2.61 | 10.75 ± 1.91 | t_(7)_= -1.000, p= n.s. |
|  | 100 μA | 9.75 ± 1.92 | 12.37 ± 1.88 | t_(7)_= -1.331, p= n.s. |
|  | 200 μA | 7.33 ± 2.50 | 5.44 ± 1.09 | t_(8)_= -0.564, p= n.s. |
|  | 400 μA | 9.14 ± 2.15 | 7.71 ± 1.84 | t_(7)_= -0.148, p= n.s. |
|  | Sham | 7.58 ± 1.72 | 6.83 ± 1.35 | t_(7)_= 0.319, p= n.s. |
| **Low-Frequency Stimulation** | 50 μA | 7.73 ± 1.19 | 7.91 ± 1.63 | t_(10)_= -0.091, p= n.s. |
|  | 100 μA | 7.58 ± 1.00 | 6.42 ± 1.22 | t_(11)_= 0.734, p= n.s. |
|  | 200 μA | 9.08 ± 1.29 | 8.00 ± 1.39 | t_(11)_= -0.564, p= n.s. |
|  | 400 μA | 11.13 ± 2.28 | 6.13 ± 1.75 | t_(7)_= 2.436, p= 0.045 |
|  | Sham | 7.58 ± 1.72 | 6.83 ± 1.35 | t_(7)_= 0.319, p= n.s. |

**(C)**

| **NOR Testing**  **(Acquisition Phase)** | **Groups** | **Exploratory Duration**  **(Mean ± S.E.M.)** | | **Effects** |
| --- | --- | --- | --- | --- |
|  |  | **Object 1** | **Object 2** |  |
| **no-HFS prior to testing** | vmPFC HFS | 12.00 ± 1.05 | 10.40 ± 0.86 | t_(9)_= 1.891, p= n.s. |
|  | Sham | 8.33 ± 0.99 | 10.33 ± 1.43 | t_(5)_= -1.142, p= n.s. |
| **HFS prior to testing** | vmPFC HFS | 8.67 ± 0.94 | 6.22 ± 0.32 | t_(8)_= 2.115, p= n.s. |
|  | Sham | 8.00 ± 1.50 | 5.43 ± 0.92 | t_(6)_= 1.090, p= n.s. |
